# ***Segue:*** Brief summaries of articles on pertinent emerging issues published elsewhere.

**DOI:** 10.3201/eid1006.040412

**Published:** 2004-06

**Authors:** Joseph E. McDade

**Affiliations:** Founding Editor of Emerging Infectious Diseases, Centers for Disease Control and Prevention, Atlanta, Georgia, USA

**Keywords:** Novel Antimicrobial Class, Influenza, Infectious Disease Archaeology, Microbial Virulence

## Novel Antimicrobial Class

Bacterial adaptation makes antibacterial drug resistance inevitable. As old medicines lose their effectiveness, scientists must find new drugs that can safely treat a broad spectrum of bacterial infections. For more than 30 years, the problem has been addressed by improving old classes of drugs. However, with this approach, we stay just ahead of the evolving bacteria; and the strategy becomes more difficult with each iteration. No truly new classes of orally active, broad-spectrum antimicrobial agents have been discovered since quinolones. A class of broad-spectrum novel ribosome inhibitors (NRI) has been found that shut down bacterial protein synthesis. Although many existing antimicrobial agents act against the ribosome, the NRIs exploit a new mechanism of action. Because bacterial populations are not familiar with NRIs, no preexisting resistance mechanisms exist in bacteria, and NRIs have consistent antimicrobial activity even against multiple drug-resistant strains.

The team conducted a comprehensive series of biologic and biochemical experiments to discover and characterize the new class, as recently reported in the journal Antimicrobial Agents and Chemotherapy. The NRIs inhibit ribosomes of both gram-positive and -negative pathogenic bacteria but will not disturb eukaryotic protein synthesis. Furthermore, the new compounds inhibit bacterial growth without toxicity to human cells, consistent with developing a new drug that kills bacteria without disturbing the human host. As further evidence of the ribosomal mechanism, the group showed that bacteria treated with NRI compounds respond by trying to overproduce ribosomal proteins. As with other ribosome inhibitors, the bacteria seem to realize that their ribosomes are failing, and they desperately try to make more to survive. In the laboratory, bacteria could be made less susceptible to NRIs by certain mutations in their ribosomes. Although, fortunately, these mutations would be difficult to generate outside the laboratory, they were useful in supporting the novel mechanism of action, since these genetic alterations did not affect the action of other ribosomal drugs. The recent data hold hope for new antimicrobial agents that can combat the rising tide of microbial resistance.

## Influenza

### Influenza Viruses with Genes from the 1918 Pandemic Virus

Eighty-six years ago, an influenza A H1N1 virus swept the globe and killed an estimated 20–40 million people. The exceptionally high death rate, especially among young adults, was not observed during later influenza pandemics of 1957 and 1968. Genetic sequence analysis of the 1918 "Spanish" influenza virus genes has not shown any features that could account for its high virulence. Therefore, we generated recombinant influenza (A/WSN/33) viruses possessing 1918 influenza gene segments to provide insights into the pathogenicity and to identify possible vaccine strategies against potentially reemergent 1918 or 1918-like viruses. Analysis of the recombinant influenza viruses points to a critical role of the 1918 hemagglutinin (HA) and neuraminidase (NA) influenza genes in virulence in the mouse model. The antigenic analysis demonstrated that the 1918 recombinant viruses most closely resembled a common influenza laboratory strain, A/Swine/Iowa/30. In fact, human survivors of the 1918 influenza pandemic had antibodies that neutralized both the 1918 HA/NA (1918 HA/NA:WSN) recombinant virus and the A/Swine/Iowa/30 virus. In studies, the protection provided by A/Swine/Iowa/30 vaccine was similar to that observed in mice that received inactivated 1918 HA/NA:WSN virus vaccine. Mice that were immune to A/Swine/Iowa/30 were protected against death and major weight loss and had undetectable virus in respiratory tissues on day 5 after virus challenge with the lethal 1918 HA/NA:WSN virus. The protection induced by A/Swine/Iowa/30 virus vaccine correlated with detectable virus–neutralizing antibodies measured in the mouse. These vaccine strategies, with data demonstrating that existing anti-influenza drugs would be effective against 1918 virus genes, provide the basis for prophylactic measures against the reemergence of new 1918-like viruses.

### Influenza Virus Tropisms

Human and avian influenza viruses target different cell types in human airway epithelium. Recent outbreaks of avian influenza infections in humans highlighted the threat of pathogenic influenza viruses emerging from a huge natural reservoir in birds. To initiate the infection, avian influenza viruses bind to cell-surface receptors containing terminal sialyl-galactosyl residues linked by 2-3-linkage, whereas human viruses, including the earliest available pandemic isolates, bind to receptors that contain terminal 2-6–linked sialyl-galactosyl residues. It is believed that a nonoptimal receptor specificity of avian viruses limits their replication in human respiratory tract and pandemic spread, but the mechanism of this restriction is not clear. Ciliated epithelium of conducting airways consists of several distinct cell types with different functions, but the roles of specific cell types in virus replication have not been defined. To investigate cellular tropism of influenza viruses, the authors employed cultures of differentiated human airway epithelial cells which closely mimic airway epithelium in vivo. The authors found that human viruses preferentially infected nonciliated cells, whereas avian viruses mainly infected ciliated cells; this pattern correlated with cell type–specific distribution of sialic acid receptors recognized by the viruses. This study suggests that two widely held concepts concerning influenza viruses (uniform susceptibility of airway epithelial cells to human viruses and a lack of receptors for avian viruses) are incorrect. These data provide insight on the emergence of pandemic viruses and open avenues for cellular studies on influenza virus replication and pathogenicity in humans.

## Infectious Disease Archaeology

### Chagas Disease

In this study, investigators reconstructed the behavior of Chagas disease (American trypanosomiasis) in the Atacama Desert over the past 9,000 years. The researchers analyzed ancient DNA to identify kinetoplast DNA of *Trypanosoma cruzi*, the disease's infectious agent transmitted by the insect vector, a triatomid bug. Specimens analyzed were from muscle and visceral tissues in excavated, naturally mummified human bodies buried in that hyperarid desert during the past 9 millennia.

Results indicated that 41% of these bodies were infected by *T. cruzi* at the time of death. Among the 11 represented populations, no statistically significant differences in prevalence rates could be demonstrated when studied by the time period, sex, or age, except for lower rates (28%) for infants. Such prevalence rates are similar to those of modern *T. cruzi*–endemic areas. These results demonstrated the well-established presence of Chagas disease in this region among wild forest animals when the first humans (the Chinchorro) arrived. By settling this region, the new arrivals initially and inadvertently exposed themselves to the triatomid bug transmitting this disease, and joined the wild animals as part of the disease's reservoir. At some undetermined time during this 9,000-year interval, a few of the vector species became adapted to the thatched roof and other features of the region's human dwellings and initiated the independent domestic cycle involving only humans and their domesticated animals. The study also suggests that, given available specimens, the history of other infectious diseases can be similarly reconstructed.

## Microbial Virulence

### Polysaccharide Intercellular Adhesin

Coagulase-negative staphylococci, with *Staphylococcus epidermidis* as the most frequently isolated species, have become the leading cause of hospital-acquired infections and infections of indwelling medical devices. In the course of these infections, biofilm formation and the ability to escape from host immune defense are regarded as the main virulence determinants. However, the factors protecting *S. epidermidis* from the immune system have remained elusive. Scientists have discovered the first specific molecule involved in immune evasion in *S. epidermidis*. The exopolysaccharide polysaccharide intercellular adhesin (PIA) was located at the cell surface of *S. epidermidis*; it protected organisms against phagocytosis by neutrophils, antibacterial peptides from human skin, and neutrophil granula. PIA was also indispensable for the formation of cellular aggregates. The positively charged PIA likely functions both as a mechanical barrier against peptides and phagocytes, and by electrostatic repulsion of the predominantly cationic antibacterial peptides. Thus, by inhibiting major mechanisms of the human innate immune defense, PIA may significantly contribute to the success of *S. epidermidis* in chronic infections. Interestingly, the genetic basis for PIA production is present in an increasing number of microorganisms, including such pathogenic species as *Yersinia pestis*, the causative agent of plague. Targeting PIA as a crucial component of both cell-cell aggregation and immune evasion processes might therefore constitute a promising way to interfere with the virulence of a series of important bacterial pathogens.

## About Segue

Segue presents brief summaries of articles on pertinent emerging issues published elsewhere and is edited by Joseph E. McDade.
